# Exploring the Multi-Faceted Role of Sirtuins in Glioblastoma Pathogenesis and Targeting Options

**DOI:** 10.3390/ijms232112889

**Published:** 2022-10-25

**Authors:** Elena Kunadis, Christina Piperi

**Affiliations:** Department of Biological Chemistry, Medical School, National and Kapodistrian University of Athens, 75 Mikras Asias Street, 11527 Athens, Greece

**Keywords:** histones, deacetylation, cell metabolism, mitochondrial regulators, stress response, DNA repair, proliferation, prognosis

## Abstract

Recent advances in glioblastoma (GB) research have shed light on the molecular characteristics, the defected intracellular signaling pathways, and the genetic and epigenetic alterations involved in their pathogenesis. Despite constant efforts, GB remains an aggressive malignant tumor with limited therapeutic approaches, poor prognosis, and a low survival rate. Emerging evidence points towards the crucial impact of epigenetic post-translational modifications in cancer development with emphasis on the regulatory role of histone deacetylation in several key cellular processes, including metabolic pathways, regulation of stress response, senescence, proliferation, DNA repair, and apoptosis. The silent information regulator proteins (Sirtuins) are deacetylases of histone and non-histone proteins that have been recently implicated in the initiation as well as in the progression of GB. Herein, we provide a critical overview of the emerging functional role and mechanism of action of the seven Sirtuins (SIRT1-7) in GB and discuss their potential targeting options in clinical practice.

## 1. Introduction

Glioblastoma (GB) represents the most common and aggressive group of neoplasms, originating from glial cells. Despite advances in treatment modalities, GB remains the most challenging malignancy to treat, with median survival varying from 9.2 to 16 months after initial diagnosis [1]. Based on available follow-up data, only 2 to 5% of GB patients survive 3 years or more and are classified as long-term survivors [2]. 

The term “glioblastoma multiforme” was originally used to describe the increased heterogeneity characterizing these tumors [3]. However, their classification was substantially revised during the past decade with integration of molecular tissue analysis in everyday practice. In the most recent 5th edition of the WHO classification of the CNS tumors, the term “multiforme” was excluded and GBs are further designated as diffuse astrocytic tumors in adults that do not harbor isocitrate dehydrogenase (IDH) mutation (IDH-wild type) but present one of the following characteristics: presence of necrosis or/and microvascular proliferation, telomerase reverse transcriptase (*TERT*) promoter mutation, and Epidermal Growth Factor Receptor (*EGFR)* gene amplification, as well as combined gain of whole chromosome 7 and loss of chromosome 10 [+7/−10] [4]. Now, GBs constitute entirely separate histological and molecular entities from IDH-mutant astrocytomas grade 2, 3, 4 [4]. From a practical standpoint, the changes that were applied on the new classification define GB as the most aggressive type that differs from IDH-mutant astrocytoma grade 4 with its dismal outcome [5]. Taken altogether, the DNA repair protein, O6-Methylguanine-DNA methyltransferase (*MGMT*) promoter methylation status remains the only prognostic factor for GB, based on the temozolomide (TMZ) treatment response of patients since MGMT methylation has been associated with longer overall survival [6].

The new WHO classification of CNS tumors highlights further the importance of molecular analysis in diagnosis, prognosis, and treatment. Current treatment options for glioblastoma remain limited to surgery, radiotherapy, and chemotherapy without addressing the genetic diversity of the tumor. Recent studies have demonstrated that epigenetic mechanisms and respective DNA or chromatin modifications play a crucial role in gliomagenesis and tumor progression, representing a valuable target for new treatment approaches [7,8]. Among the repertoire of histone modifying enzymes, histone deacetylases (HDAC) that remove acetyl groups from an ε-N-acetyl lysine amino acids on histones have been extensively investigated over the past 50 years, with several of them emerging as effective treatment targets. Eighteen HDACs have been identified thus far and classified into four classes [9]. Class III HDACs, also known as sirtuins (SIRTs) were originally investigated as potential therapeutic targets in age-related and neurodegenerative diseases [10,11], inflammatory disorders [12], muscular diseases [13], and recently, in different cancer types [14,15,16,17], including GB. The role of SIRTs as regulators of gliomagenesis is currently under thorough research with significant data. 

In this review, we summarize recent in vitro and in vivo studies focused on sirtuins’ implication in GB pathogenesis. We further address their potential mechanisms of action on GB progression, highlighting several activators and inhibitors as prospective treatment targets for future investigation.

## 2. SIRT Proteins: An Overview of Structure and Function

**Figure 1:**
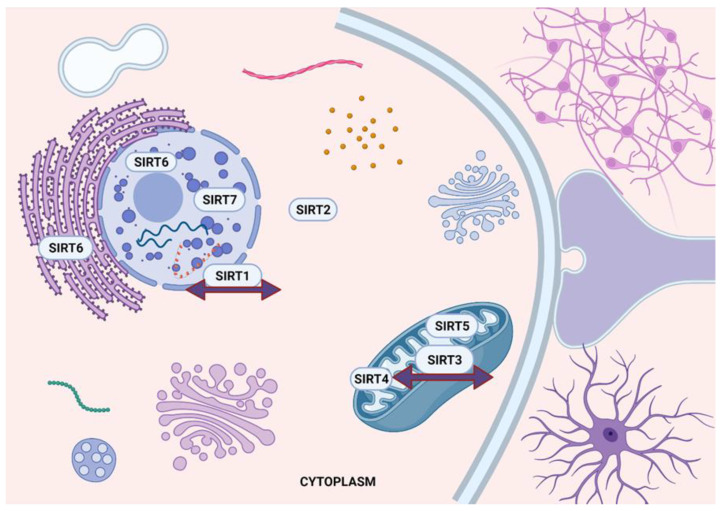
Differential localization of sirtuins (SIRT1-7) in neuronal cells. SIRT1 is mainly located in the nucleus of normal cells, whereas SIRT2 is mainly cytoplasmic. SIRT3, SIRT4, and SIRT5 are mitochondrial proteins; however, SIRT3 may also be found in the nucleus and cytosol under different cellular events. SIRT6 is a nuclear protein but can also be detected in the endoplasmic reticulum, where it inactivates NF-κΒ. SIRT7 is mainly localized in the nucleolus. (Created with BioRender.com, accessed on 17 July 2022).

The silent information regulator (SIRT) protein family was first identified in 1996, constituting seven different members (SIRT1-SIRT7) [18]. Sirtuins are considered as NAD^+^-dependent class III HDACs that regulate gene expression by altering the deacetylation-adenosine diphosphate (ADP)-ribosylation of histone and non-histone proteins [19,20]. The mammalian sirtuin family proteins vary in their cellular location (Figure 1), function and substrate action, depending on the tissue type, and metabolic and stress conditions.

Regarding their cellular localization, SIRT1, SIRT6, and SIRT7 are restricted to the nucleus, except that SIRT1 can shuttle to the cytoplasm when required, during development and in response to stress, such as during inhibition of insulin signalling [21]. SIRT6 also has been involved in the deacetylation of tumor-necrosis factor alpha (TNF-α) in the endoplasmic reticulum [22]. SIRT3, SIRT4, and SIRT5 are located in the mitochondria, regulating their function through deacetylation, demalonylation or desuccinylation activities [23]. SIRT5 exerts its enzymatic activity both in the mitochondrial and extra-mitochondrial compartments while under oxidative stress, and SIRT3 can change its location between mitochondria and nucleus [24,25]. Finally, SIRT2 can be mostly found in the cytoplasm of the central nervous system (CNS) cells, supporting its potential role in neurological disorders [26,27].

**Figure 2:**
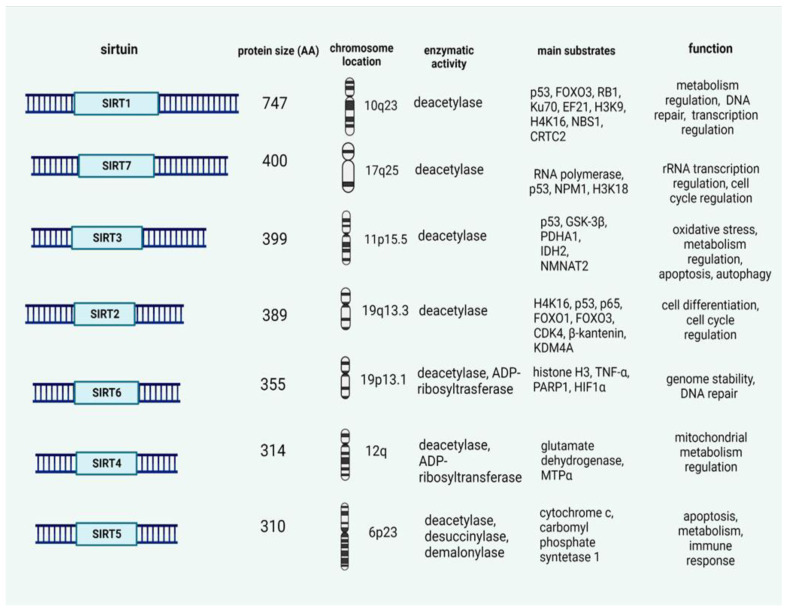
Sirtuins’ structural and functional characteristics. (Created with BioRender.com, accessed on 18 September 2022).

Based on phylogenetic analysis, SIRTs are classified into four classes, in which SIRT1, SIRT2, and SIRT3 are gathered in class I, SIRT4 into class II, SIRT 5 into class III, and SIRT6 and SIRT7 into class IV [28,29]. All sirtuin genes have conserved a core catalytic domain that contains 275 amino acids and variable N- and C-terminal domains with distinct lengths and sequence [30]. The catalytic core domain structure contains a large Rossmann fold domain, a small zinc-binding domain, and a cleft between the domains that forms the binding sites for both substrates for catalysis. All SIRT family proteins display distinct deacetylase activities and protein targets despite their conserved domain for core catalysis. Some family members exhibit additional enzymatic activities, such as ADP- ribosyltransferase and desuccinylase functions (Figure 2).

In more details, SIRT1 is the best-studied family member, mainly expressed in the nucleus where it catalyzes acetyl lysine of histone substrates and non-histone proteins controlling gene expression. SIRT1 is responsible for the regulation of cell differentiation, autophagy, apoptosis, inflammatory response, energy metabolism, and oxidative stress through activation of different cellular pathways [31,32,33,34]. Therefore, even minor fluctuations in SIRT1 expression can significantly impact cellular physiology. The first discovered non-histone target of SIRT1 is p53, the guardian of the genome and a principal tumor suppressor. SIRT1 regulates p53-mediated transcriptional activity either directly through p53 deacetylation or by deacetylating the CBP/p300 acetyltransferase, which mediates p53 acetylation [35,36]. Other transcription factors that are regulated by SIRT1 include the forkhead box (FOXO) family members, FOXO3a, FOXO1, and FOXO4, which are highly conserved proteins that control key cellular functions such as cell proliferation, differentiation, cell cycle arrest, and cell survival [37,38]. Additionally, SIRT1 regulates the transcription of various downstream inflammatory factors. Through inhibition of NF-κB signaling pathways, SIRT2 enhances oxidative metabolism and the resolution of inflammation [39,40]. Furthermore, SIRT1 interacts with several autophagy proteins such as Atg5, Atg7, and Atg8, thus synchronizing the autophagy–lysosome pathway from initiation to degradation [41,42]. 

Furthermore, the *SIRT2* gene is one of the first studied members of the family, located on autosomal chromosome 19q13.2. The SIRT2 protein is localized mainly in the cytosol [43] and is widely expressed in metabolically active tissues including kidney, heart, liver, and brain [44]. Multiple histone (H4K16, H3K18) [26] and non-histone targets (tubulin, p300, NF-κB, glucose-6-phosphate 1-dehydrogenase (G6PD), HIF1α, phosphoglycerate mutase (PGAM), IDH1 and FOXO3) have been identified [45,46,47,48]. They are involved in crucial cellular processes such as regulation of gene expression and transcription and signal transduction in metabolic pathways [49,50]. Consequently, dysregulation of SIRT2 has been associated with a broad spectrum of diseases including metabolic and neurological disorders, cancer, and aging [51,52]. By forming a crosstalk between gene expression regulation and metabolic pathways, SIRT2 is involved in all aspects of cell metabolism, including lipogenesis, fatty acid oxidation, gluconeogenesis, and glycolysis [53]. SIRT2 promotes gluconeogenesis by deacetylating FOXO1 and Phosphoenolpyruvate Carboxykinase (PEPCK), enhances glycolysis by interacting with glycolytic enzymes (ALDOA, PGK1, ENO1, GAPDH, PFKP, PKM, LDH) [54,55], and regulates insulin secretion through the PI3K/AKT pathway [56]. Furthermore, SIRT2 has been shown to promote lipid synthesis through deacetylation of ATP-citrate lyase (ACLY) [57] and inhibit it by deacetylating HNF4*α* via nuclear translocation of FOXO1. Additionally, SIRT2 can promote fatty acid oxidation by downregulating FOXO1 [58]. Moreover, it can regulate oxidative stress response through reactive oxygen species (ROS) by targeting glucose 6-phosphate dehydrogenase (G6PD), phosphoglyceride mutant enzyme (PGAM2), and NF-κB [59]. 

In turn, SIRT3 has been detected as a major mitochondrial deacetylase, being widely expressed in energy-consuming tissues such as kidney, heart, brain, and liver [60]. Most SIRT3 deacetylase targets are involved in the regulation of mitochondrial metabolism through modulation of tricarboxylic acid (TCA) and urea cycle, fatty acid oxidation, and ROS detoxification. To maintain basal adenosine triphosphate (ATP) cell levels, SIRT3 can deacetylate and activate several components of the electron transport chain (ETC), such as NDUFA9 complex I and succinate dehydrogenase (SDHA) in complex II, as well as the oligomycin-sensitivity conferring protein (OSCP) in complex V [61,62]. In addition, SIRT3 regulates the Krebs cycle through acetyl-CoA to oxaloacetate transformation [63]. SIRT3 also participates in fatty acid oxidation and amino acid catabolism. It has been shown to target enzymes involved in fatty acid oxidation regulating ATP levels during caloric restriction [64]. By facilitating conversion of Acetyl-CoA into ketones, SIRT3 reserves energy sources during fasting [65]. In respect to amino acid metabolism, SIRT3 deacetylases ornithine transcarbamylase (OTC) and therefore regulates the urea cycle [66]. Moreover, it also exerts decrotonylase activity and regulates gene expression through epigenetic mechanisms [67].

In contrast to other mitochondrial sirtuins, SIRT4 remains largely undetermined, although it is widely expressed in human organs including kidney, liver, heart, ovaries, testis, adipose tissue, muscle, and brain [68,69]. SIRT4 functions as an ADP-ribosyltransferase, and lipoamidase, but also exhibits weak deacetylase activity [70,71]. These catalytic activities implicate SIRT4 in the regulation of insulin secretion, ATP homeostasis, lipid catabolism, neurological disorders, and tumorigenesis [72,73,74]. As an ADP-ribosyltransferase, SIRT4 is responsible for the transfer of the ADP-ribosyl group from NAD^+^ to the C172 histone residue of glutamate dehydrogenase (GDH), inhibiting the metabolism of glutamine and reducing ATP production [75]. In addition to glutamine metabolism, SIRT4 is responsible for the deacetylation of malonyl-CoA-decarboxylase (MCD) and the regulation of fatty acids’ oxidation [76]. 

Among the other mitochondrial sirtuins, SIRT5 was found to exert a weak deacetylase activity but strong desuccinylase, demalonylase, and deglutarylase functions [77]. SIRT5 possesses a specific mitochondrial localization sequence (MLS) that enables its localization to the mitochondrial matrix [78], as well as to the cytosol [79]. The *SIRT5* gene encodes four SIRT5 protein isoforms (SIRT5 iso1–SIRT5 iso4). SIRT5 iso1 and SIRT5 iso2 contain an N-terminal MLS but differ in their C-terminal domain [78]. While SIRT5 isoforms are localized in mitochondria, SIRT5iso1 has been detected only in the cytosol. Two additional isoforms of SIRT5 iso3 and SIRT5 iso4 are still under investigation [80]. With the development of mass spectrometry techniques, a plethora of SIRT5 substrates have been discovered, including GAPDH, CPS1, SOD1, HMGCS2, PKM2, VLCAD, IDH2, G6PD, PDC1, and ECHA [79,81,82,83,84,85,86]. In the cytosol, SIRT5, through manolation, promotes glycolysis via GAPDH regulation [79], whereas in the mitochondrial matrix, SIRT5 regulates ammonia detoxification and the urea cycle via deacetylation of CPS1 [81]. Moreover, SIRT5 regulates isocitrate to α-ketoglutarate (α-KG) by desuccinylating isocitrate dehydrogenase 2 (IDH2), thus supporting ROS scavenging activities. In addition, SIRT5, through deglutarylation, increases the activity of glucose 6-phosphate dehydrogenase (G6PD) via the pentose phosphate pathway (PPP) and promotes NADPH production [84,87]. Rapidly proliferating cells require metabolites from PPP to synthesize ribonucleotides and regulate gene expression. G6PD expression can be deregulated in a variety of cancers and act as a regulator of viral replication [87]. Recent studies have suggested that SIRT5 plays an important role as a proviral factor necessary for the efficient replication of acute respiratory syndrome coronavirus 2 (SARS-CoV-2) through its direct interaction with the viral Nsp14 protein [88]. In summary, SIRT5 is involved in several intracellular signaling pathways, anti-inflammatory processes, cancer progression, regulation of glucose metabolism, and response to oxidative stress [85].

SIRT6 is a multifunctional protein that is highly expressed in skeletal muscles, brain, heart, thymus, and liver tissues [89,90]. The enzymatic activities of SIRT6 are not limited to deacetylation but also include defatty-acylation activity and ADP-ribosylation, thus enabling SIRT6 to regulate glucose homeostasis, DNA repair, and cellular lifespan [91]. Deacetylation of histone H3 and H4 is responsible for the compaction of chromatin, transcriptional repression, and DNA damage response [92]. The deacylase efficiency of SIRT6 has been shown to be higher compared to deacetylation activity based on the regulation of the secretion of tumor necrosis factor-α (TNF-α) by removing the fatty acyl modification on K19 and K20 [93]. Finally, SIRT6 has ADP-ribosylation activity with substrate (ADP-ribose) polymerase-1 (PARP-1) that enhances DSB repair under oxidative stress [94].

Compared to the other well-studied SIRTs, the biological function of SIRT7 is poorly defined but is gradually revealed. Its catalytic domain displays weak deacetylation activity on histones and is responsible for the highly selective deacetylation of histone H3K18, responsible for transcriptional repression, DNA damage response, and regulation of cell transformation programs [95,96]. At the same time, a wide variety of non-histone substrates of SIRT7 have been identified, including p53, NPM1, FKBP51, FOXO3, SMAD4, and PAF53 [97,98,99,100]. SIRT7-dependent deacetylation has been shown to regulate rRNA synthesis and processing via the nucleolar proteins PAF53 and U3-55k [101], and mitochondrial functions through GABPβ1 deacetylation [98]. Furthermore, SIRT7 has been reported to order the acetylation of tumor suppressor p53 [99].

The implication of SIRT proteins in diverse cellular processes and pathological conditions makes them attractive therapeutic targets. Development of specific SIRT activators and inhibitors is needed for the in-depth study of SIRTs’ function but also as potential treatment targets for a variety of diseases, such as type 2 diabetes, inflammatory and neurodegenerative disorders, and cancer. The discovery of small molecule regulators that target SIRTs has become a topic of intense interest the past 30 years with several hundred articles investigating new molecular targets as possible regulators of sirtuins’ enzymatic activity in normal cells and disease. Unfortunately, translating SIRT regulators from the bench to the clinics, faces absences of selective compounds with favorable pharmacokinetic and pharmacodynamic profiles [102,103,104,105]. Therefore, only 70 studies and respective agents have passed through the preclinical phase and were finely tested in clinical trials as potential treatment options in coronary disease, COVID-19, neurodegenerative disease, diabetes, kidney disease, and cancer [106].

## 3. Evidence of SIRTs Implication in GB Pathogenesis

A growing body of experimental evidence has implicated SIRTs in the regulation of pivotal cellular processes involved in cancer initiation and progression [14,15,16,17,106]. Although their expression is quite variable among tumor types and may act either as tumor suppressors or promoters depending on the cellular context, some SIRTs have shown clinical relevance with prognostic significance, biomarker potential, and association with drug resistance [14,15,16,17]. Emerging data have also implicated SIRTs in various aspects of glioma tumorigenesis, including proliferation, apoptosis, autophagy, stemness, angiogenesis, and metastasis, and are discussed in the following sections.

### 3.1. SIRT1 Involvement in GB Growth and Progression

SIRT1 was the first family member to be recognized as a crucial epigenetic regulator of genomic stability, aging, apoptosis, and senescence in normal cells, as well as a dual regulator in cancer cells. It has been shown to exhibit a neuroprotective role in traumatic brain injury and play an important regulatory part in CNS function. However, at the same time, SIRT1 has been implicated in the pathology of brain tumors and specifically in GB proliferation, invasion, and therapy resistance. The first documentation of SIRT1′s tumor promoter activity was demonstrated by its ability to deacetylate and inactivate tumor suppressors, such as p53, hypermethylated in cancer 1 (HIC1) and deleted in breast cancer 1 (DBC1) proteins [107,108,109]. Conversely, conditional overexpression of SIRT1 was shown to reduce the tumor burden in p53 (+/−) mice, promote genomic stability in vivo, and inhibit the NF-κB pathway, responsible for metastasis and cancer cell survival [110,111,112].

**Figure 3:**
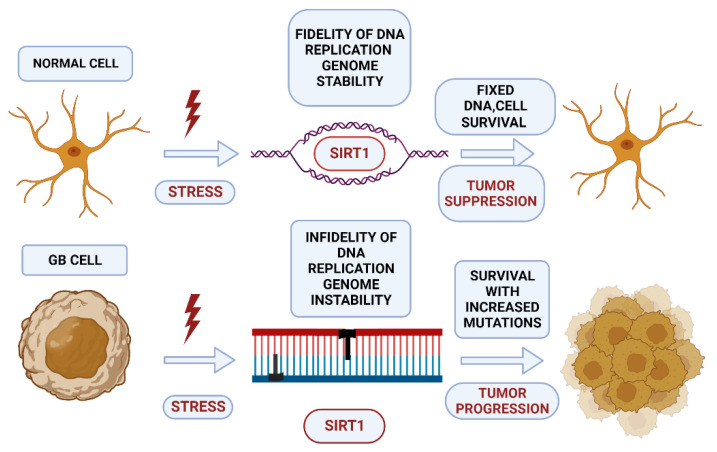
The dual role of the sirtuin SIRT1 in the GB cell. In a normal cell, stress activates SIRT1, which induces epigenetic silencing of the damaged DNA site, modulates DDR (DNA damage response), and DNA repair proteins, resulting in DNA replication fidelity and cell survival. In cancer cells and upon persistent stress signals, SIRT1 is upregulated and promotes further DNA repair with additional DNA mutations, increasing genomic instability and leading to tumor progression. (Created with BioRender.com, accessed on 17 July 2022).

Normally, SIRT1 supports cell survival of neurons exposed to oxidative stress or DNA damage, by promoting DNA repair or cell cycle arrest via induction of apoptosis. However, in cancer cells and upon persistent stress signals, SIRT1 is overexpressed and consequently promotes DNA repair, leading to additional DNA mutations, and increasing genomic instability (Figure 3). Therefore, depending on cell conditions and the stage of the disease, SIRT1 plays a regulatory role either towards tumor progression or suppression, balancing the process of oncogenesis [112,113]. 

Numerous studies have demonstrated the bifurcated role of SIRT1 protein in gliomagenesis. SIRT1 has been detected overexpressed in GB cell lines, exhibiting an essential role in both glioma proliferation and chemoresistance [114,115]. It has also been reported that overexpression of SIRT1 is associated with a shorter overall survival of patients with glioma [114]. Furthermore, downregulation of SIRT1 in GB cell lines by selective inhibitors results in suppression of the cell proliferation, migration, and angiogenesis [116,117]. However, other studies demonstrate that expression of SIRT1 is significantly downregulated in GB cell lines [118,119].

Moreover, activation of SIRT-1 was reported to induce autophagic glioma cell death and inhibit cell viability [120,121]. In neural stem cells, SIRT1 has been involved in oncogenic transformation by suppressing p53-dependent tumor surveillance, thus predicting the survival of “cancer cells with stemness” [122].

Taken altogether, the expression of SIRT1 in GB cells remains contradictory and its actual role under discussion. It is essential to detect the specific intracellular proteins involved in GB and the underlying mechanisms responsible for the inhibition or activation of SIRT1 in order to further proceed to targeted therapy. 

### 3.2. SIRT2 Implication in GB Proliferation 

**Figure 4:**
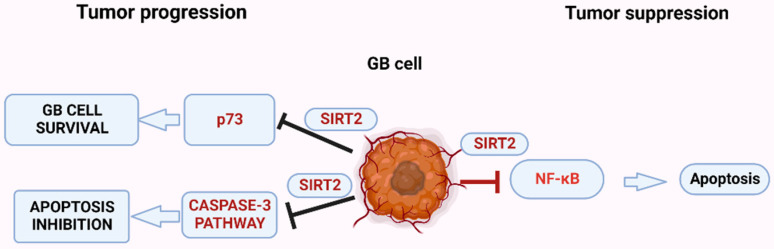
The role of the sirtuin SIRT2 in glioblastoma (GB). (Created with BioRender.com, accessed on 17 July 2022).

SIRT2 is an NAD^+^-dependent protein that deacetylates tubulin and histone H4, and modulates mitotic deposition of H4K20 methylation, resulting in cell cycle progression and genome stability [123]. SIRT2 is the most highly expressed mammalian sirtuin protein in the CNS, present primarily in the oligodendrocytes and involved in neural development [44,45]. Due to its neurodevelopmental role, SIRT2 is also involved in the pathogenesis of various neurodegenerative disorders, including Parkinson’s, Alzheimer’s, and Huntington’s diseases [124,125]. The *SIRT2* gene location is at the 19q13.2 chromosomal region, which is often altered in GB [126]. A study by Hiratsuka et al. reported that SIRT2 expression was downregulated in glioma tissues and cell lines, while its overexpression was shown to decrease GB cell proliferation and survival [126]. Conversely, a study by He et al. revealed that downregulation of SIRT2 activity can induce necrosis and apoptosis in C6 glioma cells [127]. Similar conclusions were made by a study by Funato et al., showing that SIRT2 is required for the tumorigenicity of GB cells by regulating the transcriptional activity of tumor suppressor p73, while knockdown of SIRT2 induced cell-cycle arrest (Figure 4) [128]. These differential results could be attributed to the use of the C6 rat glioma cell line, which differs from the human glioma cells, as well as to the use of a 2D culture model that is not representative of the in vivo tumor microenvironment. At the study by Funato et al., a neurospheres assay-3D model was used, which is regarded as the “gold standard” for in vitro GB stem cell population culture [128]. Unfortunately, this assay has its own disadvantages, allowing cells to create their own niche, with mixed populations of cells and a small number of genuine stem cells [129]. Moreover, in other malignancies such as breast cancer, SIRT2 expression is closely correlated with the stage of the disease, with a significant tumor suppressive role during early carcinogenesis and controversial effects in the advanced phase of the disease [130]. Based on this evidence, and on the findings of dual expression of SIRT1 in GB, it is suggested that SIRT2 expression is also dependent on the disease stage, and its activation/suppression mechanisms need to be further investigated.

### 3.3. SIRT3 Implication in GB Cell Metabolism

Cancer is a highly energy-consuming process, and reprogramming of cellular metabolism is a crucial hallmark of tumorigenesis. SIRT3 is a major mitochondrial NAD-dependent deacetylase that is involved in the regulation of mitochondrial metabolism and energy cell homeostasis [131]. It has been associated to the pathogenesis of cancer, DM, neurodegeneration, and cardiological and liver diseases [132]. 

In GB, aberrant regulation of several metabolic pathways has been suggested to contribute to their infiltrative phenotype, enhancing tumor growth. To support the high metabolic demands of GB, glucose uptake is increased through deregulation of carbohydrate cell metabolism. This metabolic reprogramming, known as the Warburg effect, is actively controlled by SIRT3. In other malignancies, it has been shown that loss of SIRT3 upregulates hypoxia-inducible factor 1-alpha (HIF1α) target genes, resulting in stimulation of cell proliferation and increased cell glucose uptake [133]. Additionally, SIRT3 deacetylates the isocitrate dehydrogenase 2 (IDH2), protecting cells from oxidative stress and regulating reactive oxygen species (ROS) homeostasis [134]. IDH2 is frequently mutated in secondary GB (70–75%) [135] and is a biomarker of a good prognosis. 

**Figure 5:**
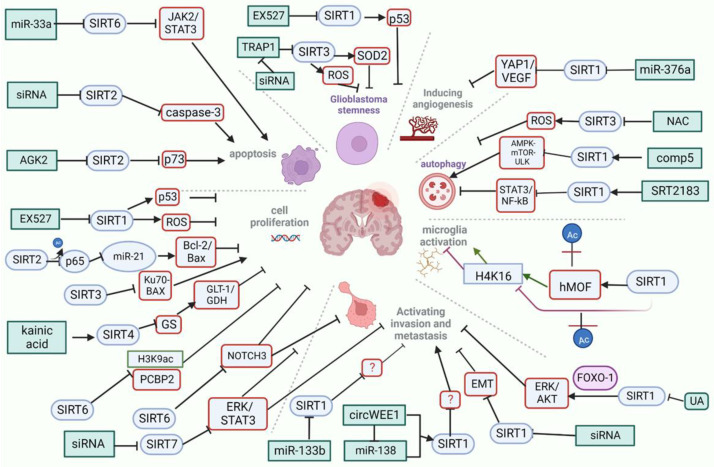
Overview of sirtuins-associated signaling pathways implicated in glioblastoma development and progression. Red rectangle: signaling pathways; blue oval: sirtuin member; blue rectangle: sirtuin targeting agent or approach. (Created with BioRender.com, accessed on 16 October 2022).

According to a study by Luo et al., SIRT3 expression is upregulated in glioma tissues and is correlated with tumor aggressiveness (according to WHO grade) and prognosis [136]. Patients with low SIRT3 expression were shown to exhibit better prognosis and overall survival. Further investigation revealed that SIRT3 overexpression downregulated glioma cell apoptosis via the Ku70–BAX pathway (Figure 5) [136]. Additionally, Park et al. studied the interplay between TRAP1 mitochondrial chaperone and SIRT3 in GSC and demonstrated that inactivation of TRAP1 or SIRT3 leads to loss of stemness and suppression of GSC formation [137]. 

Altogether, SIRT3 as a main player of mitochondrial energy homeostasis may contribute to metabolic alterations in GB cells and represents a possible therapeutic target that needs further exploration for GB treatment.

### 3.4. SIRT4 Involvement in GB Cell Metabolism

SIRT4 is a mitochondrial sirtuin with mostly ADP-ribosylation activity, but little deacetylase activity [138]. SIRT4 is highly expressed in the brain tissue, especially in astrocytes and radial glia during embryogenesis, and its expression decreases throughout development [139]. The amino acid glutamine is a key nutrient of the brain tissue and, along with glutamate, plays a crucial role in normal brain function. During gliomagenesis, cancer cells that grow in a glutamate–glutamine-rich microenvironment try to reprogram the glutamine metabolism to support their growth and activity [140]. Excitotoxicity refers to the presence of a large amount of glutamate outside the cell that under normal conditions regulates astrocyte metabolism through glutamate transporter 1 (GLT-1). SIRT4 controls glutamine metabolism via regulation of glutamate dehydrogenase (GDH) activity. Inhibition of GDH by SIRT4 results in downregulation of glutamate metabolism and reduces ATP production [141]. A study by Yalcin et al. showed that by regulating glutamate metabolism, SIRT4 prevents excitotoxicity in glioma cells and acts as a GB suppressor [142].

It is therefore suggested that targeting the glutamate metabolic pathway through SIRT4 regulation could be a possible effective approach to inhibit gliomagenesis progression (Figure 5) and needs further investigation.

### 3.5. Role of SIRT5 in GB Progression

SIRT5 is the third mitochondrial enzyme with low deacetylation activity [143] but with strong desuccinylation, demalonylation, and deglutarylation activities [144]. SIRT5 tissue expression is modest in the brain, heart, and liver cells, as well as in lymphoblasts [21,145,146]. The main cellular processes regulated by SIRT5 are urea metabolism, through activation of carbamoyl phosphate synthetase 1 (CPS1) [147], and oxidative stress via ROS regulation [148]. Additionally, SIRT5 interacts with p53 and suppresses transcriptional activity via succinylation of p53 at lysine 120 (K120) [144]. Despite research evidence supporting a high biological impact of SIRT5 on various cellular processes, data on the role of SIRT5 in GB onset are scarce. Chen et al. demonstrated that SIRT5 expression is significantly downregulated in GB and is correlated with worse prognosis and overall survival. Moreover, the expression levels of SIRT5 were linked to DNA methylation activity in GB [149].

It is evident that mitochondrial SIRTs play a crucial role in a variety of metabolic processes related to GB progression and pathogenesis. While research on mitochondrial SIRTs in GB is still in progress, several studies indicate that these proteins may exhibit a dual role, acting both as tumor suppressors and tumor promoters. Modulation of this balance may play a critical role in targeted therapy approaches for GB, which are greatly needed. 

### 3.6. SIRT6 Implication in GB Growth

SIRT6 is predominantly a nuclear enzyme that is highly expressed in muscle, brain, and heart tissues [150]. Due to its location, SIRT6 appears to be involved mainly in the regulation of nuclear processes through histone deacetylation. SIRT6 binds to the telomeres and modulates their function through deacetylation of H3 lysine 9 (H3K9) and lysine 56 (H3K56) [151]. SIRT6 deacetylation regulates the transcriptional activity via the NF-κB, HIF1, and c-myc pathways, downregulating gene expression [152]. The substrates of SIRT6 include, in addition to multiple H3 and H4 histone lysine residues, non-histone proteins. SIRT6 can directly deacetylate the K549 of histone GCN5 and enhance its enzymatic activity in the regulation of hepatic gluconeogenesis [153]. Furthermore, SIRT6 can remove the acetyl groups from the pyruvate kinase muscle isozyme (PKM2) lysine 433 residue, activating its nuclear export and inhibiting PKM2 oncogenic activity [154]. In addition to the well-established deacetylase activity, SIRT6 possesses an efficient defatty-acylase activity that is involved in the regulation of several proteins’ secretion. TNF-α, a proinflammation cytokine, was one of the first targets of SIRT6 deacylation to be studied. SIRT6 has been demonstrated to promote the secretion of TNF-α, activating apoptosis and cell-survival signaling pathways [93]. Furthermore, SIRT6 is also a mono-ADP-ribosylation enzyme that can regulate the activity of poly ADP-ribose polymerase-1 (PARP-1), enhancing double strand break (DSB) repair under oxidative stress and maintaining genomic stability [94]. To date, SIRT6 has been proposed as a unique enzyme that modulates gene expression and cell metabolism, promotes DNA repair, and prolongs lifespan. SIRT6 mainly acts as a tumor suppressor and regulator of age-related pathological changes [155]. It is an important regulator of cancer progression and is reported to be associated with the prognosis of cancer patients. A meta-analysis conducted by Wu et al., including data from 1577 patients, revealed that low expression of SIRT6 may predict a favorable survival rate for patients with solid tumors [15]. 

Although the role of SIRT6 in GB is still under investigation, the study by Chang et al. showed that SIRT6 is downregulated and negatively correlated with miR-33 expression in GB cell lines. Moreover, SIRT6 overexpression was shown to reduce cell survival and proliferation of GB cells [156]. Another study demonstrated that SIRT6 regresses GB cell growth through induction of apoptosis and suppression of the JAK2/STAT3 signaling pathway (Figure 5) [157]. Downregulation of the NOTCH3 pathway by SIRT6 has been detected, confirming the oncosuppressive role of SIRT6 in GB [158]. 

### 3.7. SIRT7 Implication in GB Progression

SIRT7 is present in eukaryotes, located mainly in the nucleus, where it is involved in multiple pathways that regulate rRNA processing and protein translation [101,159]. SIRT7 activity is necessary for the regulation of rDNA transcription during mitosis [160] and is known to play a role in protein synthesis through reduction of tRNA levels [161]. SIRT6 and SIRT7 share many interacting proteins that are involved in chromatin remodeling, DNA repair, and aging processes [162]. Several studies have demonstrated a role of SIRT7 in hepatic lipid metabolism, in the regulation of the ubiquitin pathway [163]. Although several studies have investigated the role of SIRT7 in disease, particularly in cancer, indicating an oncogenic function [164,165], its possible role in GB is unclear and demands further study. Mu et al. demonstrated that SIRT7 was upregulated in GB tissues and correlated with tumor aggression [166]. In addition, SIRT7 expression can serve as a prognostic biomarker for GB, as its downregulation was shown to decrease expression of cyclin-dependent kinase 2 (CDK2) and STAT3, leading to suppression of GB cell proliferation and invasion in vitro (Figure 5) [166].

Further research studies have shown that SIRT7 enhances *IDH1* transcription, and that SIRT7 insufficiency downregulates IDH1 expression, resulting in cell metabolic reprogramming during tumorigenesis [167]. IDH1 mRNA and protein levels were found elevated in primary GB supporting the aggressive tumor growth and therapy resistance, while IDH1 inactivation promoted apoptosis and enhanced response to targeted therapies [168]. Further investigation of the potential targeting of SIRT7-IDH1 axis as a possible novel therapeutic tool for GB is necessary. According to the studies investigating SIRT3-IDH2 regulation and the SIRT7-IDH1 axis, a combined downregulation of the SIRT3–SIRT7 proteins could possibly be effective, with IDH presenting a specific target in both secondary and primary GB, independent of IDH mutation. 

## 4. Conclusions

During the past 50 years, the involvement of SIRTs in cancer biology has been studied, and the accumulated evidence reveals their regulatory role in several important steps during the development and progression of different tumor types [103,169,170,171,172]. SIRTs are involved in various biological pathways in which they could act as either tumor suppressors or activators, depending on the stage of the disease, the histological type, and the tumor microenvironment [173]. Although the biological functions of SIRTs are still under investigation, a plethora of possible inhibitors (SIRTi) and activators (STACs) that may directly target specific SIRTs or their underlying pathways are already under evaluation. The design of new SIRT-targeted molecules is constantly demanded to help elucidate the role of SIRTs in cancer development and the associated mechanisms of tumorigenesis, with the prospect of introducing novel SIRT regulators into clinical practice, as promising treatment options. To date, most SIRTi apart from nicotinamide are grouped according to their pharmacophore types, which include β-napthol (sirtinol, splitomicin, cambinol), indole (EX-527), urea (suramin) and thiourea derivatives (tenovins), as well as some miscellaneous compounds (pan-SIRTi MC2494). However, limited evidence is available regarding their specific anti-cancer activities [106,114,116,128].

Regarding STACS, the first that were discovered were phenolic compounds, such as Resveratrol and other polyphenols, quercetin, butein, fisetin, piceatannol, and isoliquiritigenin. Recent efforts have further revealed some non-polyphenolic STACs, such as the new second generation (SRT1460, SRT1720, SRT2183) and third generation (STAC-5, STAC-9, and STAC-10) small molecules, with their anti-cancer activity being under investigation [106,117,121]. 

An increasing number of studies indicate that SIRTs activity is involved in GB pathology (Figure 5). Although many questions regarding the role of specific SIRTs in GB remain unanswered or are still under investigation, there is a growing interest in this area, mainly due to the urgent need for new treatment strategies for these tumors. Treatment recommendations for GB have remained unchanged since 2005 and offer only a median patient overall survival of 15 months [174]. New molecular regulators targeting SIRTs in GB have demonstrated favorable results in vivo and in vitro (Table 1). Unfortunately, these do not translate into immediate success in clinical practice because of multiple factors, including tumor specificity and heterogeneity, stemness, blood–brain barrier drug penetration capacity, and interactions with the microenvironment [175,176,177]. At present, none of the SIRT inhibitors/activators are under clinical trial for GB [178]. More than 50 clinical trials, however, are ongoing for SIRT targeting therapy for aging or age-related disorders, including neurodegenerative and cardiovascular diseases, as well as DM. Evidence of the therapeutic value of SIRT-targeted therapy in other disorders, along with success in the in vitro/in vivo GB studies, will provide the impetus for novel prospective studies in the field of GB research and treatment.

## Figures and Tables

**Table 1:** Studies implicating the sirtuins (SIRT1-7) in GB pathogenesis and indicative targeting approaches.

Sirtuin Type	Cellular Location	In Vitro/In Vivo Models	SIRT Targeting Agent/Approach	Role of SIRT in GB and Mechanisms of Regulation	Reference
**SIRT1**	Nucleus/ cytoplasm	U251 and U87MG cell lines, in vivo	SIRT1 silenced by shRNA, EX527 inhibitor	SIRT1 expression is upregulated in glioma tissues and cell lines. Patients with higher SIRT1 expression exhibit poorer prognosis. SIRT1 inhibition sensitizes glioma cells to temozolomide (TMZ) treatment in vitro and in vivo	[114]
		U251 and U118 MG cell lines, in vivo		Downregulation of SIRT1 blocks inhibitory effects of urolithin A on proliferation and migration of GB cells and modifies expression level of FOXO1	[179]
		A172, LN229, U251, and U373 cell lines, in vivo	miR-376a, selective SIRT1 inhibitor	miR-376a directly targets and inhibits SIRT1 in GB via downregulation of the YAP1 and VEGF signalling axis resulting in suppression of proliferation, migration, and angiogenesis abilities of GB cells.	[180]
		U87 and U251 cell lines	SIRT1-small interfering RNA (siRNA)	SIRT1 positively associated with viability and invasion of GB cells via epithelial-mesenchymal transition	[115]
		Microglia BV2 GB C6, GL261 rodent cell lines	-	Overexpression of SIRT1 promotes the increase in H4K16 acetylation level in cell, and modulates microglial pro-tumoral activation	[181]
		U87MG, A172 and LN229 Cell lines	EX-527, SIRT1 inhibitor	SIRT1 inhibition produced a decreased expression of stemness markers –Sox-2 and Oct-4 and decreased capacity to form gliomaspheres (compared with control cells).	[116]
		HT1080, U251, and HEK293T cell lines; patient derived glioma lines MGG152, MGG119, MGG18, MGG123	EX-527, Sirt inhibitor	Activation of SIRT1 can selectively target IDH-mutant tumors	[182]
		U87MG and T98G cell lines	SRT2183, SIRT activator	SRT2183 suppresses GB cell growth by cell cycle arrest and apoptosis via SIRT activation and upregulation of Bim and downregulation of Bcl-2 and Bcl-xL	[117]
		U87MG and T98G cells lines, in vivo	F0911-7667 (Comp 5) SIRT activator	F0911-7667 induces autophagic cell death/mitophagy in GB cells through SIRT1 upregulation	[121]
		GB cell lines (U251, U87, T98-G) and human brain normal glial cell lines (HEB), in vivo	circWEE1 SIRT activator	CircWEE1 induces GB cell growth by upregulating the expression of SIRT1 through sponge adsorption of miR-138	[120]
		Human GB U87 cell line	SIRT1 as a downstream effector miR-133b	miR-133b overexpression significantly inhibits GB cell proliferation. SIRT1 overexpression reverses the miR-133b-mediated suppression of GB cell proliferation and invasion.	[183]
**SIRT2**	Cytoplasm	GB cell lines T98G, U87MG, A172, U251, and CCFSTTG1		SIRT2 expression is downregulated in human GB tissues and cell lines. SIRT2 regresses glioma growth via the NF-κB–p21-apoptosis axis. SIRT2 overexpression decreases cell proliferation and colony formation capacity	[126,184]
		G201, G301, G302 G402, G405		SIRT2 acts as a tumor suppressor gene in human gliomas. SIRT2 overexpression reduces colony formation of human glioma cells.	[184]
		GB1–GB13 and GB16	SIRT2-specific inhibitor AGK2, siRNA	Downregulation of SIRT2 results in significant inhibition of GB sphere formation, and in growth arrest and apoptosis of GB cells	[128]
		C6 GB cells		SIRT2 promotes survival maintenance of glioma cells. Inhibition of SIRT2 activity can induce necrosis apoptosis in GB cells	[127]
**SIRT3**	Mitochondria	GB tissues, U87 and U251 glioma cell lines		SIRT3 is upregulated in GB tissues. Low SIRT3 expression shows better prognosis in GB then high SIRT3 expression	[136]
		GB tissues, LN229, T98G, U87 and U251, in vivo	siRNA	SIRT3 deacetylates TRAP1. Inactivation of TRAP1 or SIRT3 leads to loss of stemness, and suppression of tumor formation in GB stem cells	[137]
		Human GB cell lines T98G and LN229	siRNA	SIRT3 is a positive regulator of autophagy; inhibition of SIRT3 down regulates autophagy induced by hypoxia in GB	[185]
**SIRT4**	Mitochondria	A172 cell line		SIRT4 overexpression increases cell viability after kainic acid treatment. SIRT4 decreases cell death by preventing excitotoxicity.	[142]
**SIRT5**	Mitochondria			SIRT5 expression is decreased in GB tissues. SIRT5 acts as a tumor suppressor. SIRT 5 shows promise as a prognostic biomarker in GB	[149]
**SIRT6**	Nucleus	Human GB cell lines, U87, T98, A172 and U251		SIRT6 is downregulated and inversely correlated with miR-33a expression in GB. Deregulation of miR-33a may promote tumor development in human GB by regulating the expression of SIRT6	[156]
		GB cell lines U251, U87, LN18, and A172, and normal human astrocyte cell	siRNA	SIRT6 is downregulated in GB. Knockdown of SIRT6 increased cell proliferation, migration, and invasion of cancer cells	[158]
		U87-MG and T98G and one human normal glial cell line HEB		SIRT6 overexpression promoted apoptosis, reduced oxidative stress, and suppressed the activation of the JAK2/STAT3 signaling pathway in GB	[157]
		GB cell lines T98G		PCBP2 expression is inhibited by SIRT6. SIRT6 inhibits GB cell proliferation and colony formation in vitro and glioma cell growth in vivo in a PCBP2 dependent manner	[186]
**SIRT7**	Nucleus	U87 and U251 cell line	siRNA	SIRT7 is upregulated in human GB tissues and high expression of SIRT7 is positively correlated with GB malignancy. SIRT7 induces GB cell proliferation and invasion by activation of the ERK/STAT3 signalling pathway	[166]
		U-118 MG, U87, and U251	siRNA	*SIRT7* knockdown reduced IDH1 protein and mRNA levels GB cell lines	[167]

## Data Availability

Not applicable.

## References

[1] Brown N.F., Ottaviani D., Tazare J., Gregson J., Kitchen N., Brandner S., Fersht N., Mulholland P. (2022). Survival Outcomes and Prognostic Factors in Glioblastoma. Cancers.

[2] Ramos-Fresnedo A., Pullen M.W., Perez-Vega C., Domingo R.A., Akinduro O.O., Almeida J.P., Suarez-Meade P., Marenco-Hillembrand L., Jentoft M.E., Bendok B.R. (2022). The Survival Outcomes of Molecular Glioblastoma IDH-Wildtype: A Multicenter Study. J. Neuro-Oncol..

[3] Louis D.N., Perry A., Reifenberger G., von Deimling A., Figarella-Branger D., Cavenee W.K., Ohgaki H., Wiestler O.D., Kleihues P., Ellison D.W. (2016). The 2016 World Health Organization Classification of Tumors of the Central Nervous System: A Summary. Acta Neuropathol..

[4] Louis D.N., Perry A., Wesseling P., Brat D.J., Cree I.A., Figarella-Branger D., Hawkins C., Ng H.K., Pfister S.M., Reifenberger G. (2021). The 2021 WHO Classification of Tumors of the Central Nervous System: A Summary. Neuro-Oncology.

[5] Stoyanov G.S., Lyutfi E., Georgieva R., Georgiev R., Dzhenkov D.L., Petkova L., Ivanov B.D., Kaprelyan A., Ghenev P. (2022). Reclassification of Glioblastoma Multiforme According to the 2021 World Health Organization Classification of Central Nervous System Tumors: A Single Institution Report and Practical Significance. Cureus.

[6] Binabaj M.M., Bahrami A., ShahidSales S., Joodi M., Joudi Mashhad M., Hassanian S.M., Anvari K., Avan A. (2018). The Prognostic Value of MGMT Promoter Methylation in Glioblastoma: A Meta-Analysis of Clinical Trials. J. Cell. Physiol..

[7] Kunadis E., Lakiotaki E., Korkolopoulou P., Piperi C. (2021). Targeting Post-Translational Histone Modifying Enzymes in Glioblastoma. Pharmacol. Ther..

[8] Waldmann T., Schneider R. (2013). Targeting Histone Modifications—Epigenetics in Cancer. Curr. Opin. Cell Biol..

[9] Seto E., Yoshida M. (2014). Erasers of Histone Acetylation: The Histone Deacetylase Enzymes. Cold Spring Harb. Perspect. Biol..

[10] Yeong K.Y., Berdigaliyev N., Chang Y. (2020). Sirtuins and Their Implications in Neurodegenerative Diseases from a Drug Discovery Perspective. ACS Chem. Neurosci..

[11] Lagunas-Rangel F.A. (2022). SIRT7 in the Aging Process. Cell. Mol. Life Sci..

[12] Mendes K.L., de Farias Lelis D., Santos S.H.S. (2017). Nuclear sirtuins and inflammatory signaling pathways. Cytokine Growth Factor Rev..

[13] Kuno A., Horio Y. (2016). SIRT1: A Novel Target for the Treatment of Muscular Dystrophies. Oxidative Med. Cell. Longev..

[14] Zhao Q.Y., Zhou J., Li F., Guo S., Zhang L., Li J., Qi Q., Shi Y. (2022). The Role and Therapeutic Perspectives of Sirtuin 3 in Cancer Metabolism Reprogramming, Metastasis, and Chemoresistance. Front. Oncol..

[15] Wu X., Wang S., Zhao X., Lai S., Yuan Z., Zhan Y., Ni K., Liu Z., Liu L., Xin R. (2022). Clinicopathological and Prognostic Value of SIRT6 in Patients with Solid Tumors: A Meta-Analysis and TCGA Data Review. Cancer Cell Int..

[16] Mahjabeen I., Rizwan M., Fareen G., Waqar Ahmed M., Farooq Khan A., Akhtar Kayani M. (2022). Mitochondrial Sirtuins Genetic Variations and Gastric Cancer Risk: Evidence from Retrospective Observational Study. Gene.

[17] Yao L., Wang Y. (2022). Bioinformatic Analysis of the Effect of the Sirtuin Family on Differentiated Thyroid Carcinoma. BioMed Res. Int..

[18] Trzebiatowski J.R., Escalante-Semerena J.C. (1997). Purification and Characterization of CobT, the Nicotinate-Mononucleotide:5,6-Dimethylbenzimidazole Phosphoribosyltransferase Enzyme from Salmonella Typhimurium LT2. J. Biol. Chem..

[19] Glozak M.A., Sengupta N., Zhang X., Seto E. (2005). Acetylation and Deacetylation of Non-Histone Proteins. Gene.

[20] Martínez-Redondo P., Vaquero A. (2013). The Diversity of Histone versus Nonhistone Sirtuin Substrates. Genes Cancer.

[21] Michishita E., Park J.Y., Burneskis J.M., Barrett J.C., Horikawa I. (2005). Evolutionarily Conserved and Nonconserved Cellular Localizations and Functions of Human SIRT Proteins. Mol. Biol. Cell.

[22] Michishita E., McCord R.A., Berber E., Kioi M., Padilla-Nash H., Damian M., Cheung P., Kusumoto R., Kawahara T.L.A., Barrett J.C. (2008). SIRT6 Is a Histone H3 Lysine 9 Deacetylase That Modulates Telomeric Chromatin. Nature.

[23] He W., Newman J.C., Wang M.Z., Ho L., Verdin E. (2012). Mitochondrial Sirtuins: Regulators of Protein Acylation and Metabolism. Trends Endocrinol. Metab..

[24] Osborne B., Bentley N.L., Montgomery M.K., Turner N. (2016). The role of mitochondrial sirtuins in health and disease. Free Radic. Biol. Med..

[25] Michan S., Sinclair D. (2007). Sirtuins in Mammals: Insights into Their Biological Function. Biochem. J..

[26] Vaquero A., Scher M.B., Dong H.L., Sutton A., Cheng H.L., Alt F.W., Serrano L., Sternglanz R., Reinberg D. (2006). SirT2 Is a Histone Deacetylase with Preference for Histone H4 Lys 16 during Mitosis. Genes Dev..

[27] Chen X., Lu W., Wu D. (2021). Sirtuin 2 (SIRT2): Confusing Roles in the Pathophysiology of Neurological Disorders. Front. Neurosci..

[28] Frye R.A. (2000). Phylogenetic Classification of Prokaryotic and Eukaryotic Sir2-like Proteins. Biochem. Biophys. Res. Commun..

[29] Frye R.A. (2006). Evolution of Sirtuins from Archaea to Vertebrates. Histone Deacetylases.

[30] Moniot S., Weyand M., Steegborn C. (2012). Structures, Substrates, and Regulators of Mammalian Sirtuins—Opportunities and Challenges for Drug Development. Front. Pharmacol..

[31] Thakur B.L., Baris A.M., Fu H., Redon C.E., Pongor L.S., Mosavarpour S., Gross J.M., Jang S.M., Sebastian R., Utani K. (2022). Convergence of SIRT1 and ATR Signaling to Modulate Replication Origin Dormancy. Nucleic Acids Res..

[32] Zhang S., Sun S., Wei X., Zhang M., Chen Y., Mao X., Chen G., Liu C. (2022). Short-Term Moderate Caloric Restriction in a High-Fat Diet Alleviates Obesity via AMPK/SIRT1 Signaling in White Adipocytes and Liver. Food Nutr. Res..

[33] Yang Y., Liu Y., Wang Y., Chao Y., Zhang J., Jia Y., Tie J., Hu D. (2022). Regulation of SIRT1 and Its Roles in Inflammation. Front. Immunol..

[34] Higgins C.B., Mayer A.L., Zhang Y., Franczyk M., Ballentine S., Yoshino J., DeBosch B.J. (2022). SIRT1 Selectively Exerts the Metabolic Protective Effects of Hepatocyte Nicotinamide Phosphoribosyltransferase. Nat. Commun..

[35] Lee J.T., Gu W. (2013). SIRT1: Regulator of P53 Deacetylation. Genes Cancer.

[36] Solomon J.M., Pasupuleti R., Xu L., McDonagh T., Curtis R., DiStefano P.S., Huber L.J. (2006). Inhibition of SIRT1 Catalytic Activity Increases P53 Acetylation but Does Not Alter Cell Survival Following DNA Damage. Mol. Cell. Biol..

[37] van der Horst A., Tertoolen L.G.J., de Vries-Smits L.M.M., Frye R.A., Medema R.H., Burgering B.M.T. (2004). FOXO4 Is Acetylated upon Peroxide Stress and Deacetylated by the Longevity Protein HSir2SIRT1. J. Biol. Chem..

[38] Giannakou M.E., Partridge L. (2004). The Interaction between FOXO and SIRT1: Tipping the Balance towards Survival. Trends Cell Biol..

[39] Yang H., Zhang W., Pan H., Feldser H.G., Lainez E., Miller C., Leung S., Zhong Z., Zhao H., Sweitzer S. (2012). SIRT1 Activators Suppress Inflammatory Responses through Promotion of P65 Deacetylation and Inhibition of NF-ΚB Activity. PLoS ONE.

[40] Kauppinen A., Suuronen T., Ojala J., Kaarniranta K., Salminen A. (2013). Antagonistic Crosstalk between NF-ΚB and SIRT1 in the Regulation of Inflammation and Metabolic Disorders. Cell. Signal..

[41] Ou X., Lee M.R., Huang X., Messina-Graham S., Broxmeyer H.E. (2014). SIRT1 Positively Regulates Autophagy and Mitochondria Function in Embryonic Stem Cells under Oxidative Stress. Stem Cells.

[42] Kim J.Y., Mondaca-Ruff D., Singh S., Wang Y. (2022). SIRT1 and Autophagy: Implications in Endocrine Disorders. Front. Endocrinol..

[43] North B.J., Verdin E. (2004). Sirtuins: Sir2-Related NAD-Dependent Protein Deacetylases. Genome Biol..

[44] Maxwell M.M., Tomkinson E.M., Nobles J., Wizeman J.W., Amore A.M., Quinti L., Chopra V., Hersch S.M., Kazantsev A.G. (2011). The Sirtuin 2 Microtubule Deacetylase Is an Abundant Neuronal Protein That Accumulates in the Aging CNS. Hum. Mol. Genet..

[45] Wang Y., Yang J., Hong T., Chen X., Cui L. (2019). SIRT2: Controversy and Multiple Roles in Disease and Physiology. Ageing Res. Rev..

[46] Nakagawa T., Guarente L. (2011). Sirtuins at a Glance. J. Cell Sci..

[47] Wang F., Chan C.H., Chen K., Guan X., Lin H.K., Tong Q. (2012). Deacetylation of FOXO3 by SIRT1 or SIRT2 Leads to Skp2-Mediated FOXO3 Ubiquitination and Degradation. Oncogene.

[48] Wang B., Ye Y., Yang X., Liu B., Wang Z., Chen S., Jiang K., Zhang W., Jiang H., Mustonen H. (2020). SIRT 2-dependent IDH 1 Deacetylation Inhibits Colorectal Cancer and Liver Metastases. EMBO Rep..

[49] Rothgiesser K.M., Erener S., Waibel S., Lüscher B., Hottiger M.O. (2010). SIRT2 Regulates NF-ΚB-Dependent Gene Expression through Deacetylation of P65 Lys310. J. Cell Sci..

[50] Singh C.K., Chhabra G., Ndiaye M.A., Garcia-Peterson L.M., MacK N.J., Ahmad N. (2018). The Role of Sirtuins in Antioxidant and Redox Signaling. Antioxid. Redox Signal..

[51] Sola-Sevilla N., Ricobaraza A., Hernandez-Alcoceba R., Aymerich M.S., Tordera R.M., Puerta E. (2021). Understanding the Potential Role of Sirtuin 2 on Aging: Consequences of SIRT2.3 Overexpression in Senescence. Int. J. Mol. Sci..

[52] Manjula R., Anuja K., Alcain F.J. (2021). SIRT1 and SIRT2 Activity Control in Neurodegenerative Diseases. Front. Pharmacol..

[53] Zhu C., Dong X., Wang X., Zheng Y., Qiu J., Peng Y., Xu J., Chai Z., Liu C. (2022). Multiple Roles of SIRT2 in Regulating Physiological and Pathological Signal Transduction. Genet. Res..

[54] Cha Y., Han M.J., Cha H.J., Zoldan J., Burkart A., Jung J.H., Jang Y., Kim C.H., Jeong H.C., Kim B.G. (2017). Metabolic Control of Primed Human Pluripotent Stem Cell Fate and Function by the MiR-200c–SIRT2 Axis. Nat. Cell Biol..

[55] Hamaidi I., Zhang L., Kim N., Wang M.H., Iclozan C., Fang B., Liu M., Koomen J.M., Berglund A.E., Yoder S.J. (2020). Sirt2 Inhibition Enhances Metabolic Fitness and Effector Functions of Tumor-Reactive T Cells. Cell Metab..

[56] Ramakrishnan G., Davaakhuu G., Kaplun L., Chung W.C., Rana A., Atfi A., Miele L., Tzivion G. (2014). Sirt2 Deacetylase Is a Novel AKT Binding Partner Critical for AKT Activation by Insulin. J. Biol. Chem..

[57] Lin R., Tao R., Gao X., Li T., Zhou X., Guan K.L., Xiong Y., Lei Q.Y. (2013). Acetylation Stabilizes ATP-Citrate Lyase to Promote Lipid Biosynthesis and Tumor Growth. Mol. Cell.

[58] Park S.Y., Chung M.J., Son J.Y., Yun H.H., Park J.M., Yim J.H., Jung S.J., Lee S.H., Jeong K.S. (2021). The Role of Sirtuin 2 in Sustaining Functional Integrity of the Liver. Life Sci..

[59] de Angelis M., Amatore D., Checconi P., Zevini A., Fraternale A., Magnani M., Hiscott J., de Chiara G., Palamara A.T., Nencioni L. (2022). Influenza Virus Down-Modulates G6PD Expression and Activity to Induce Oxidative Stress and Promote Its Replication. Front. Cell. Infect. Microbiol..

[60] Jin L., Galonek H., Israelian K., Choy W., Morrison M., Xia Y., Wang X., Xu Y., Yang Y., Smith J.J. (2009). Biochemical Characterization, Localization, and Tissue Distribution of the Longer Form of Mouse SIRT3. Protein Sci..

[61] Ahn B.H., Kim H.S., Song S., In H.L., Liu J., Vassilopoulos A., Deng C.X., Finkel T. (2008). A Role for the Mitochondrial Deacetylase Sirt3 in Regulating Energy Homeostasis. Proc. Natl. Acad. Sci. USA.

[62] Finley L.W.S., Haas W., Desquiret-Dumas V., Wallace D.C., Procaccio V., Gygi S.P., Haigis M.C. (2011). Succinate Dehydrogenase Is a Direct Target of Sirtuin 3 Deacetylase Activity. PLoS ONE.

[63] Hallows W.C., Lee S., Denu J.M. (2006). Sirtuins Deacetylate and Activate Mammalian Acetyl-CoA Synthetases. Proc. Natl. Acad. Sci. USA.

[64] Sebastian C., Mostoslavsky R. (2010). SIRT3 in Calorie Restriction: Can You Hear Me Now?. Cell.

[65] Shi L., Tu B.P. (2015). Acetyl-CoA and the Regulation of Metabolism: Mechanisms and Consequences. Curr. Opin. Cell Biol..

[66] Shimazu T., Hirschey M.D., Hua L., Dittenhafer-Reed K.E., Schwer B., Lombard D.B., Li Y., Bunkenborg J., Alt F.W., Denu J.M. (2010). SIRT3 Deacetylates Mitochondrial 3-Hydroxy-3-Methylglutaryl CoA Synthase 2 and Regulates Ketone Body Production. Cell Metab..

[67] Bao X., Wang Y., Li X., Li X.M., Liu Z., Yang T., Wong C.F., Zhang J., Hao Q., Li X.D. (2014). Identification of “erasers” for Lysine Crotonylated Histone Marks Using a Chemical Proteomics Approach. eLife.

[68] Wei W., Hu P., Qin M., Chen G., Wang F., Yao S., Jin M., Xie Z., Zhang X. (2022). SIRT4 Is Highly Expressed in Retinal Müller Glial Cells. Front. Neurosci..

[69] SIRT4 Sirtuin 4 [Homo Sapiens (Human)]—Gene—NCBI. https://www.ncbi.nlm.nih.gov/gene/23409.

[70] Laurent G., German N.J., Saha A.K., de Boer V.C.J., Davies M., Koves T.R., Dephoure N., Fischer F., Boanca G., Vaitheesvaran B. (2013). SIRT4 Coordinates the Balance between Lipid Synthesis and Catabolism by Repressing Malonyl CoA Decarboxylase. Mol. Cell.

[71] Tomaselli D., Steegborn C., Mai A., Rotili D. (2020). Sirt4: A Multifaceted Enzyme at the Crossroads of Mitochondrial Metabolism and Cancer. Front. Oncol..

[72] Li J., Zhan H., Ren Y., Feng M., Wang Q., Jiao Q., Wang Y., Liu X., Zhang S., Du L. (2022). Sirtuin 4 Activates Autophagy and Inhibits Tumorigenesis by Upregulating the P53 Signaling Pathway. Cell Death Differ..

[73] Chakrabarty R.P., Chandel N.S. (2022). Beyond ATP, New Roles of Mitochondria. Biochemist.

[74] Li J., Yan H., Xiang R., Yang W., Ye J., Yin R., Yang J., Chi Y. (2022). ATP Secretion and Metabolism in Regulating Pancreatic Beta Cell Functions and Hepatic Glycolipid Metabolism. Front. Physiol..

[75] Argmann C., Auwerx J. (2006). Insulin Secretion: SIRT4 Gets in on the Act. Cell.

[76] Ji Z., Liu G.H., Qu J. (2022). Mitochondrial Sirtuins, Metabolism, and Aging. J. Genet. Genom..

[77] Du J., Zhou Y., Su X., Yu J.J., Khan S., Jiang H., Kim J., Woo J., Kim J.H., Choi B.H. (2011). Sirt5 Is a NAD-Dependent Protein Lysine Demalonylase and Desuccinylase. Science.

[78] Matsushita N., Yonashiro R., Ogata Y., Sugiura A., Nagashima S., Fukuda T., Inatome R., Yanagi S. (2011). Distinct Regulation of Mitochondrial Localization and Stability of Two Human Sirt5 Isoforms. Genes Cells.

[79] Nishida Y., Rardin M.J., Carrico C., He W., Sahu A.K., Gut P., Najjar R., Fitch M., Hellerstein M., Gibson B.W. (2015). SIRT5 Regulates Both Cytosolic and Mitochondrial Protein Malonylation with Glycolysis as a Major Target. Mol. Cell.

[80] Kumar S., Lombard D.B. (2018). Functions of the Sirtuin Deacylase SIRT5 in Normal Physiology and Pathobiology. Crit. Rev. Biochem. Mol. Biol..

[81] Nakagawa T., Lomb D.J., Haigis M.C., Guarente L. (2009). SIRT5 Deacetylates Carbamoyl Phosphate Synthetase 1 and Regulates the Urea Cycle. Cell.

[82] Lin Z.F., Xu H.B., Wang J.Y., Lin Q., Ruan Z., Liu F.B., Jin W., Huang H.H., Chen X. (2013). SIRT5 Desuccinylates and Activates SOD1 to Eliminate ROS. Biochem. Biophys. Res. Commun..

[83] Rardin M.J., He W., Nishida Y., Newman J.C., Carrico C., Danielson S.R., Guo A., Gut P., Sahu A.K., Li B. (2013). SIRT5 Regulates the Mitochondrial Lysine Succinylome and Metabolic Networks. Cell Metab..

[84] Zhou L., Wang F., Sun R., Chen X., Zhang M., Xu Q., Wang Y., Wang S., Xiong Y., Guan K.-L. (2016). SIRT5 Promotes IDH2 Desuccinylation and G6PD Deglutarylation to Enhance Cellular Antioxidant Defense. EMBO Rep..

[85] Wang Y., Chen H., Zha X. (2022). Overview of SIRT5 as a Potential Therapeutic Target: Structure, Function and Inhibitors. Eur. J. Med. Chem..

[86] Sadhukhan S., Liu X., Ryu D., Nelson O.D., Stupinski J.A., Li Z., Chen W., Zhang S., Weiss R.S., Locasale J.W. (2016). Metabolomics-Assisted Proteomics Identifies Succinylation and SIRT5 as Important Regulators of Cardiac Function. Proc. Natl. Acad. Sci. USA.

[87] Meng Q., Zhang Y., Hao S., Sun H., Liu B., Zhou H., Wang Y., Xu Z.-X. (2022). Recent Findings in the Regulation of G6PD and Its Role in Diseases. Front. Pharmacol..

[88] Walter M., Chen I.P., Vallejo-Gracia A., Kim I.-J., Bielska O., Lam V.L., Hayashi J.M., Cruz A., Shah S., Gross J.D. (2022). SIRT5 Is a Proviral Factor That Interacts with SARS-CoV-2 Nsp14 Protein. bioRxiv.

[89] Uhlén M., Fagerberg L., Hallström B.M., Lindskog C., Oksvold P., Mardinoglu A., Sivertsson Å., Kampf C., Sjöstedt E., Asplund A. (2015). Tissue-Based Map of the Human Proteome. Science.

[90] Li Y., Jin J., Wang Y. (2022). SIRT6 Widely Regulates Aging, Immunity, and Cancer. Front. Oncol..

[91] Pastor B.M., Mostoslavsky R. (2016). SIRT6: A New Guardian of Mitosis. Nat. Struct. Mol. Biol..

[92] Wang W.W., Zeng Y., Wu B., Deiters A., Liu W.R. (2016). A Chemical Biology Approach to Reveal Sirt6-Targeted Histone H3 Sites in Nucleosomes. ACS Chem. Biol..

[93] Jiang H., Khan S., Wang Y., Charron G., He B., Sebastian C., Du J., Kim R., Ge E., Mostoslavsky R. (2013). SIRT6 Regulates TNF-α Secretion through Hydrolysis of Long-Chain Fatty Acyl Lysine. Nature.

[94] Mao Z., Hine C., Tian X., van Meter M., Au M., Vaidya A., Seluanov A., Gorbunova V. (2011). SIRT6 Promotes DNA Repair under Stress by Activating PARP1. Science.

[95] Barber M.F., Michishita-Kioi E., Xi Y., Tasselli L., Kioi M., Moqtaderi Z., Tennen R.I., Paredes S., Young N.L., Chen K. (2012). SIRT7 Links H3K18 Deacetylation to Maintenance of Oncogenic Transformation. Nature.

[96] Vazquez B.N., Thackray J.K., Simonet N.G., Kane-Goldsmith N., Martinez-Redondo P., Nguyen T., Bunting S., Vaquero A., Tischfield J.A., Serrano L. (2016). SIRT7 Promotes Genome Integrity and Modulates Non-Homologous End Joining DNA Repair. EMBO J..

[97] Karim M.F., Yoshizawa T., Sobuz S.U., Sato Y., Yamagata K. (2017). Sirtuin 7-Dependent Deacetylation of DDB1 Regulates the Expression of Nuclear Receptor TR4. Biochem. Biophys. Res. Commun..

[98] Ryu D., Jo Y.S., lo Sasso G., Stein S., Zhang H., Perino A., Lee J.U., Zeviani M., Romand R., Hottiger M.O. (2014). A SIRT7-Dependent Acetylation Switch of GABPβ1 Controls Mitochondrial Function. Cell Metab..

[99] Vakhrusheva O., Smolka C., Gajawada P., Kostin S., Boettger T., Kubin T., Braun T., Bober E. (2008). Sirt7 Increases Stress Resistance of Cardiomyocytes and Prevents Apoptosis and Inflammatory Cardiomyopathy in Mice. Circ. Res..

[100] Blank M.F., Grummt I. (2017). The Seven Faces of SIRT7. Transcription.

[101] Chen S., Blank M.F., Iyer A., Huang B., Wang L., Grummt I., Voit R. (2016). SIRT7-Dependent Deacetylation of the U3-55k Protein Controls Pre-RRNA Processing. Nat. Commun..

[102] Dai H., Sinclair D.A., Ellis J.L., Steegborn C. (2018). Sirtuin Activators and Inhibitors: Promises, Achievements, and Challenges. Pharmacol. Ther..

[103] Fiorentino F., Mautone N., Menna M., D’Acunzo F., Mai A., Rotili D. (2022). Sirtuin Modulators: Past, Present, and Future Perspectives. Futur. Med. Chem..

[104] Villalba J.M., Alcaín F.J. (2012). Sirtuin Activators and Inhibitors. Biofactors.

[105] Curry A.M., White D.S., Donu D., Cen Y. (2021). Human Sirtuin Regulators: The “Success” Stories. Front. Physiol..

[106] Carafa V., Altucci L., Nebbioso A. (2019). Dual Tumor Suppressor and Tumor Promoter Action of Sirtuins in Determining Malignant Phenotype. Front. Pharmacol..

[107] Zhao W., Kruse J.P., Tang Y., Jung S.Y., Qin J., Gu W. (2008). Negative Regulation of the Deacetylase SIRT1 by DBC1. Nature.

[108] Wen Y.C., Wang D.H., RayWhay C.Y., Luo J., Gu W., Baylin S.B. (2005). Tumor Suppressor HIC1 Directly Regulates SIRT1 to Modulate P53-Dependent DNA-Damage Responses. Cell.

[109] Yeung F., Hoberg J.E., Ramsey C.S., Keller M.D., Jones D.R., Frye R.A., Mayo M.W. (2004). Modulation of NF-KappaB-Dependent Transcription and Cell Survival by the SIRT1 Deacetylase. EMBO J..

[110] Oberdoerffer P., Michan S., McVay M., Mostoslavsky R., Vann J., Park S.K., Hartlerode A., Stegmuller J., Hafner A., Loerch P. (2008). SIRT1 Redistribution on Chromatin Promotes Genomic Stability but Alters Gene Expression during Aging. Cell.

[111] Herranz D., Muñoz-Martin M., Cañamero M., Mulero F., Martinez-Pastor B., Fernandez-Capetillo O., Serrano M. (2010). Sirt1 Improves Healthy Ageing and Protects from Metabolic Syndrome-Associated Cancer. Nat. Commun..

[112] Zhao L., Cao J., Hu K., He X., Yun D., Tong T., Han L. (2020). Sirtuins and Their Biological Relevance in Aging and Age-Related Diseases. Aging Dis..

[113] Chen H., Lin R., Zhang Z., Wei Q., Zhong Z., Huang J., Xu Y. (2019). Sirtuin 1 Knockdown Inhibits Glioma Cell Proliferation and Potentiates Temozolomide Toxicity via Facilitation of Reactive Oxygen Species Generation. Oncol. Lett..

[114] Li Y., Chen X., Cui Y., Wei Q., Chen S., Wang X. (2019). Effects of SIRT1 Silencing on Viability, Invasion and Metastasis of Human Glioma Cell Lines. Oncol. Lett..

[115] Nissan N.E., Saad A.M., Elshewy K.M., Al-Husseini M.J., Alfaar A.S., Patricia D., July J., Wahjoepramono E.J., Prayogi G., Wuisan Z.G. (2017). SIRT1 Inhibition Exhibits Decreased Pluripotency in Cancer Stem Cells of Glioma. Ann. Oncol..

[116] Ye T., Wei L., Shi J., Jiang K., Xu H., Hu L., Kong L., Zhang Y., Meng S., Piao H. (2019). Sirtuin1 Activator SRT2183 Suppresses Glioma Cell Growth Involving Activation of Endoplasmic Reticulum Stress Pathway. BMC Cancer.

[117] Lages E., Guttin A., el Atifi M., Ramus C., Ipas H., Dupré I., Rolland D., Salon C., Godfraind C., deFraipont F. (2011). MicroRNA and Target Protein Patterns Reveal Physiopathological Features of Glioma Subtypes. PLoS ONE.

[118] Romeo Giovanna S., Conti A., Polito F., Tomasello C., Barresi V., la Torre D., Cucinotta M., Angileri Filippo F., Bartolotta M., di Giorgio Maria R. (2016). MiRNA Regulation of Sirtuin-1 Expression in Human Astrocytoma. Oncol. Lett..

[119] Guo J., Yu L. (2021). CircWEE1/MiR-138 Axis Promotes the Malignant Progression of Glioma by Regulating SIRT1. Transl. Cancer Res..

[120] Yao Z.Q., Zhang X., Zhen Y., He X.Y., Zhao S., Li X.F., Yang B., Gao F., Guo F.Y., Fu L. (2018). A Novel Small-Molecule Activator of Sirtuin-1 Induces Autophagic Cell Death/Mitophagy as a Potential Therapeutic Strategy in Glioblastoma. Cell Death Dis..

[121] Lee J.-S., Park J.-R., Kwon O.-S., Lee T.-H., Nakano I., Miyoshi H., Chun K.-H., Park M.-J., Lee H.J., Kim S.U. (2015). SIRT1 Is Required for Oncogenic Transformation of Neural Stem Cells and for the Survival of “Cancer Cells with Neural Stemness” in a P53-Dependent Manner. Neuro-Oncology.

[122] North B.J., Marshall B.L., Borra M.T., Denu J.M., Verdin E. (2003). The Human Sir2 Ortholog, SIRT2, Is an NAD+-Dependent Tubulin Deacetylase. Mol. Cell.

[123] Li W., Zhang B., Tang J., Cao Q., Wu Y., Wu C., Guo J., Ling E.A., Liang F. (2007). Sirtuin 2, a Mammalian Homolog of Yeast Silent Information Regulator-2 Longevity Regulator, Is an Oligodendroglial Protein That Decelerates Cell Differentiation through Deacetylating Alpha-Tubulin. J. Neurosci..

[124] Biella G., Fusco F., Nardo E., Bernocchi O., Colombo A., Lichtenthaler S.F., Forloni G., Albani D. (2016). Sirtuin 2 Inhibition Improves Cognitive Performance and Acts on Amyloid-β Protein Precursor Processing in Two Alzheimer’s Disease Mouse Models. J. Alzheimer’s Dis..

[125] Ahmed Rasheed B.K., Wiltshire R.N., Bigner S.H., Bigner D.D. (1999). Molecular Pathogenesis of Malignant Gliomas. Curr. Opin. Oncol..

[126] Hiratsuka M., Inoue T., Toda T., Kimura N., Shirayoshi Y., Kamitani H., Watanabe T., Ohama E., Tahimic C.G.T., Kurimasa A. (2003). Proteomics-Based Identification of Differentially Expressed Genes in Human Gliomas: Down-Regulation of SIRT2 Gene. Biochem. Biophys. Res. Commun..

[127] He X., Nie H., Hong Y., Sheng C., Xia W., Ying W. (2012). SIRT2 Activity Is Required for the Survival of C6 Glioma Cells. Biochem. Biophys. Res. Commun..

[128] Funato K., Hayashi T., Echizen K., Negishi L., Shimizu N., Koyama-Nasu R., Nasu-Nishimura Y., Morishita Y., Tabar V., Todo T. (2018). SIRT2-Mediated Inactivation of P73 Is Required for Glioblastoma Tumorigenicity. EMBO Rep..

[129] Bez A., Corsini E., Curti D., Biggiogera M., Colombo A., Nicosia R.F., Pagano S.F., Parati E.A. (2003). Neurosphere and Neurosphere-Forming Cells: Morphological and Ultrastructural Characterization. Brain Res..

[130] Zhang L., Kim S., Ren X. (2020). The Clinical Significance of SIRT2 in Malignancies: A Tumor Suppressor or an Oncogene?. Front. Oncol..

[131] Lombard D.B., Alt F.W., Cheng H.-L., Bunkenborg J., Streeper R.S., Mostoslavsky R., Kim J., Yancopoulos G., Valenzuela D., Murphy A. (2007). Mammalian Sir2 Homolog SIRT3 Regulates Global Mitochondrial Lysine Acetylation. Mol. Cell Biol..

[132] Ouyang S., Zhang Q., Lou L., Zhu K., Li Z., Liu P., Zhang X. (2022). The Double-Edged Sword of SIRT3 in Cancer and Its Therapeutic Applications. Front. Pharmacol..

[133] Finley L.W.S., Carracedo A., Lee J., Souza A., Egia A., Zhang J., Teruya-Feldstein J., Moreira P.I., Cardoso S.M., Clish C.B. (2011). SIRT3 Opposes Reprogramming of Cancer Cell Metabolism through HIF1α Destabilization. Cancer Cell.

[134] Yu W., Dittenhafer-Reed K.E., Denu J.M. (2012). SIRT3 Protein Deacetylates Isocitrate Dehydrogenase 2 (IDH2) and Regulates Mitochondrial Redox Status. J. Biol. Chem..

[135] Krell D., Assoku M., Galloway M., Mulholland P., Tomlinson I., Bardella C. (2011). Screen for IDH1, IDH2, IDH3, D2HGDH and L2HGDH Mutations in Glioblastoma. PLoS ONE.

[136] Luo K., Huang W., Tang S. (2018). Sirt3 Enhances Glioma Cell Viability by Stabilizing Ku70–BAX Interaction. Onco Targets Ther..

[137] Park H.K., Hong J.H., Oh Y.T., Kim S.S., Yin J., Lee A.J., Chae Y.C., Kim J.H., Park S.H., Park C.K. (2019). Interplay between TRAP1 and Sirtuin-3 Modulates Mitochondrial Respiration and Oxidative Stress to Maintain Stemness of Glioma Stem Cells. Cancer Res..

[138] Pannek M., Simic Z., Fuszard M., Meleshin M., Rotili D., Mai A., Schutkowski M., Steegborn C. (2017). Crystal Structures of the Mitochondrial Deacylase Sirtuin 4 Reveal Isoform-Specific Acyl Recognition and Regulation Features. Nat. Commun..

[139] Komlos D., Mann K.D., Zhuo Y., Ricupero C.L., Hart R.P., Liu A.Y.C., Firestein B.L. (2013). Glutamate Dehydrogenase 1 and SIRT4 Regulate Glial Development. Glia.

[140] Natarajan S.K., Venneti S. (2019). Glutamine Metabolism in Brain Tumors. Cancers.

[141] Jeong S.M., Xiao C., Finley L.W.S., Lahusen T., Souza A.L., Pierce K., Li Y.H., Wang X., Laurent G., German N.J. (2013). SIRT4 Has Tumor Suppressive Activity and Regulates the Cellular Metabolic Response to DNA Damage by Inhibiting Mitochondrial Glutamine Metabolism. Cancer Cell.

[142] Yalçın G.D., Colak M. (2020). SIRT4 Prevents Excitotoxicity via Modulating Glutamate Metabolism in Glioma Cells. Hum. Exp. Toxicol..

[143] Schuetz A., Min J., Antoshenko T., Wang C.L., Allali-Hassani A., Dong A., Loppnau P., Vedadi M., Bochkarev A., Sternglanz R. (2007). Structural Basis of Inhibition of the Human NAD+-Dependent Deacetylase SIRT5 by Suramin. Structure.

[144] Liu X., Rong F., Tang J., Zhu C., Chen X., Jia S., Wang Z., Sun X., Deng H., Zha H. (2021). Repression of P53 Function by SIRT5-Mediated Desuccinylation at Lysine 120 in Response to DNA Damage. Cell Death Differ..

[145] Brain Tissue Expression of SIRT5—Summary—The Human Protein Atlas. https://www.proteinatlas.org/ENSG00000124523-SIRT5/brain.

[146] Tissue Expression of SIRT5—Staining in Heart Muscle—The Human Protein Atlas. https://v17.proteinatlas.org/ENSG00000124523-SIRT5/tissue/heart+muscle.

[147] Ogura M., Nakamura Y., Tanaka D., Zhuang X., Fujita Y., Obara A., Hamasaki A., Hosokawa M., Inagaki N. (2010). Overexpression of SIRT5 Confirms Its Involvement in Deacetylation and Activation of Carbamoyl Phosphate Synthetase 1. Biochem. Biophys. Res. Commun..

[148] Liu B., Che W., Zheng C., Liu W., Wen J., Fu H., Tang K., Zhang J., Xu Y. (2013). SIRT5: A Safeguard against Oxidative Stress-Induced Apoptosis in Cardiomyocytes. Cell. Physiol. Biochem..

[149] Chen X., Xu Z., Zeng S., Wang X., Liu W., Qian L., Wei J., Yang X., Shen Q., Gong Z. (2019). SIRT5 Downregulation Is Associated with Poor Prognosis in Glioblastoma. Cancer Biomark..

[150] Liszt G., Ford E., Kurtev M., Guarente L. (2005). Mouse Sir2 Homolog SIRT6 Is a Nuclear ADP-Ribosyltransferase. J. Biol. Chem..

[151] Polyakova O., Borman S., Grimley R., Vamathevan J., Hayes B., Solari R. (2012). Identification of Novel Interacting Partners of Sirtuin6. PLoS ONE.

[152] Zwaans B.M.M., Lombard D.B. (2014). Interplay between Sirtuins, MYC and Hypoxia-Inducible Factor in Cancer-Associated Metabolic Reprogramming. Dis. Model. Mech..

[153] Dominy J.E., Lee Y., Jedrychowski M.P., Chim H., Jurczak M.J., Camporez J.P., Ruan H.-B., Feldman J., Pierce K., Mostoslavsky R. (2012). The Deacetylase Sirt6 Activates the Acetyltransferase GCN5 and Suppresses Hepatic Gluconeogenesis. Mol. Cell.

[154] Bhardwaj A., Das S. (2016). SIRT6 Deacetylates PKM2 to Suppress Its Nuclear Localization and Oncogenic Functions. Proc. Natl. Acad. Sci. USA.

[155] Chang A.R., Ferrer C.M., Mostoslavsky R. (2020). SIRT6, a Mammalian Deacylase with Multitasking Abilities. Physiol. Rev..

[156] Chang M., Qiao L., Li B., Wang J., Zhang G., Shi W., Liu Z., Gu N., Di Z., Wang X. (2017). Suppression of SIRT6 by MiR-33a Facilitates Tumor Growth of Glioma through Apoptosis and Oxidative Stress Resistance. Oncol. Rep..

[157] Feng J., Yan P.F., Zhao H.Y., Zhang F.C., Zhao W.H., Feng M. (2016). SIRT6 Suppresses Glioma Cell Growth via Induction of Apoptosis, Inhibition of Oxidative Stress and Suppression of JAK2/STAT3 Signaling Pathway Activation. Oncol. Rep..

[158] Chen X., Li D., Gao Y., Cao Y., Hao B. (2018). Histone Deacetylase SIRT6 Inhibits Glioma Cell Growth through Down-Regulating NOTCH3 Expression. Acta Biochim. Biophys. Sin..

[159] Wang W.W., Angulo-Ibanez M., Lyu J., Kurra Y., Tong Z., Wu B., Zhang L., Sharma V., Zhou J., Lin H. (2019). A Click Chemistry Approach Reveals the Chromatin-Dependent Histone H3K36 Deacylase Nature of SIRT7. J. Am. Chem. Soc..

[160] Grob A., Roussel P., Wright J.E., McStay B., Hernandez-Verdun D., Sirri V. (2009). Involvement of SIRT7 in Resumption of RDNA Transcription at the Exit from Mitosis. J. Cell Sci..

[161] Tsai Y.C., Greco T.M., Cristea I.M. (2014). Sirtuin 7 Plays a Role in Ribosome Biogenesis and Protein Synthesis. Mol. Cell. Proteom..

[162] Lee N., Kim D.K., Kim E.S., Park S.J., Kwon J.H., Shin J., Park S.M., Moon Y.H., Wang H.J., Gho Y.S. (2014). Comparative Interactomes of SIRT6 and SIRT7: Implication of Functional Links to Aging. Proteomics.

[163] Yoshizawa T., Karim M.F., Sato Y., Senokuchi T., Miyata K., Fukuda T., Go C., Tasaki M., Uchimura K., Kadomatsu T. (2014). SIRT7 Controls Hepatic Lipid Metabolism by Regulating the Ubiquitin-Proteasome Pathway. Cell Metab..

[164] Zhang S., Chen P., Huang Z., Hu X., Chen M., Hu S., Hu Y., Cai T. (2015). Sirt7 Promotes Gastric Cancer Growth and Inhibits Apoptosis by Epigenetically Inhibiting MiR-34a. Sci. Rep..

[165] Zhang C., Li Y., Liu B., Ning C., Li Y., Wang Y., Li Z. (2022). Discovery of SIRT7 Inhibitor as New Therapeutic Options Against Liver Cancer. Front. Cell Dev. Biol..

[166] Mu P., Liu K.U.N., Lin Q., Yang W., Liu D.A.N., Lin Z., Shao W.E.I., Ji T. (2019). Sirtuin 7 Promotes Glioma Proliferation and Invasion through Activation of the ERK/STAT3 Signaling Pathway. Oncol. Lett..

[167] Su F., Tang X., Li G., Koeberle A., Liu B. (2021). SIRT7–SREBP1 Restrains Cancer Cell Metabolic Reprogramming by Upregulating IDH1. Genome Instab. Dis..

[168] Calvert A.E., Chalastanis A., Wu Y., Hurley L.A., Kouri F.M., Bi Y., Kachman M., May J.L., Bartom E., Hua Y. (2017). Cancer-Associated IDH1 Promotes Growth and Resistance to Targeted Therapies in the Absence of Mutation. Cell Rep..

[169] Zhao E., Hou J., Ke X., Abbas M.N., Kausar S., Zhang L., Cui H. (2019). The Roles of Sirtuin Family Proteins in Cancer Progression. Cancers.

[170] Edatt L., Poyyakkara A., Raji G.R., Ramachandran V., Shankar S.S., Kumar V.B.S. (2020). Role of Sirtuins in Tumor Angiogenesis. Front. Oncol..

[171] Jaiswal A., Xudong Z., Zhenyu J., Saretzki G. (2022). Mitochondrial Sirtuins in Stem Cells and Cancer. FEBS J..

[172] Costa-Machado L.F., Fernandez-Marcos P.J. (2019). The Sirtuin Family in Cancer. Cell Cycle.

[173] Carafa V., Rotili D., Forgione M., Cuomo F., Serretiello E., Hailu G.S., Jarho E., Lahtela-Kakkonen M., Mai A., Altucci L. (2016). Sirtuin functions and modulation: From chemistry to the clinic. Clin. Epigenet..

[174] Delgado-López P.D., Corrales-García E.M. (2016). Survival in Glioblastoma: A Review on the Impact of Treatment Modalities. Clin. Transl. Oncol..

[175] Shergalis A., Bankhead A., Luesakul U., Muangsin N., Neamati N. (2018). Current Challenges and Opportunities in Treating Glioblastomas. Pharmacol. Rev..

[176] Noch E.K., Ramakrishna R., Magge R. (2018). Challenges in the Treatment of Glioblastoma: Multisystem Mechanisms of Therapeutic Resistance. World Neurosurg..

[177] Habashy K.J., Mansour R., Moussalem C., Sawaya R., Massaad M.J. (2022). Challenges in Glioblastoma Immunotherapy: Mechanisms of Resistance and Therapeutic Approaches to Overcome Them. Br. J. Cancer.

[178] Search of: Sirt|Glioblastoma—List Results—ClinicalTrials.Gov. https://clinicaltrials.gov/ct2/results?cond=Glioblastoma&term=sirt&cntry=&state=&city=&dist=.

[179] Liu C.L., Zhao D., Li J.J., Liu S., An J.J., Wang D., Hu F.A., Qiu C.Y., Cui M.H. (2022). Inhibition of Glioblastoma Progression by Urolithin A in Vitro and in Vivo by Regulating Sirt1-FOXO1 Axis via ERK/AKT Signaling Pathways. Neoplasma.

[180] Deng Y.W., Shu Y.G., Sun S.L. (2022). MiR-376a Inhibits Glioma Proliferation and Angiogenesis by Regulating YAP1/VEGF Signalling via Targeting of SIRT1. Transl. Oncol..

[181] Saidi D., Cheray M., Osman A.M., Stratoulias V., Lindberg O.R., Shen X., Blomgren K., Joseph B. (2017). Glioma-Induced SIRT1-Dependent Activation of HMOF Histone H4 Lysine 16 Acetyltransferase in Microglia Promotes a Tumor Supporting Phenotype) Glioma-Induced SIRT1-Dependent Activation of HMOF Histone H4 Lysine 16 Acetyltransferase in Microglia Promotes a Tumor Supporting Phenotype. OncoImmunology.

[182] Miller J.J., Fink A., Banagis J.A., Nagashima H., Subramanian M., Lee C.K., Melamed L., Tummala S.S., Tateishi K., Wakimoto H. (2021). Sirtuin Activation Targets IDH-Mutant Tumors. Neuro-Oncology.

[183] Li C., Liu Z., Yang K., Chen X., Zeng Y., Liu J., Li Z., Liu Y., Li C., Liu Z. (2016). MiR-133b Inhibits Glioma Cell Proliferation and Invasion by Targeting Sirt1. Oncotarget.

[184] Li Y., Dai D., Lu Q., Fei M., Li M., Wu X. (2013). Sirt2 Suppresses Glioma Cell Growth through Targeting NF-ΚB-MiR-21 Axis. Biochem. Biophys. Res. Commun..

[185] Qiao A., Wang K., Yuan Y., Guan Y., Ren X., Li L., Chen X., Li F., Chen A.F., Zhou J. (2016). Sirt3-Mediated Mitophagy Protects Tumor Cells against Apoptosis under Hypoxia. Oncotarget.

[186] Chen X., Hao B., Liu Y., Dai D., Han G., Li Y., Wu X., Zhou X., Yue Z., Wang L. (2014). The Histone Deacetylase SIRT6 Suppresses the Expression of the RNA-Binding Protein PCBP2 in Glioma. Biochem. Biophys. Res. Commun..

